# MicroRNA-101-3p Suppresses mTOR and Causes Mitochondrial Fragmentation and Cell Degeneration in COPD

**DOI:** 10.1155/2022/5933324

**Published:** 2022-12-05

**Authors:** Lei Fang, Xinggang Wang, Ming Zhang, Petra Khan, Michael Tamm, Michael Roth

**Affiliations:** ^1^Departments of Biomedicine & Internal Medicine, University and University Hospital Basel, Basel, Switzerland; ^2^Reproductive Medicine Centre, Shanghai Ninth People's Hospital, Shanghai Jiaotong University, Shanghai, China; ^3^Department of Respiratory and Critical Care Medicine, The Second Affiliated Hospital of Xi'an Jiaotong University, Xi'an, Shaanxi, China

## Abstract

**Background:**

Cigarette smoke is assumed to cause the loss of airway wall structure in chronic obstructive pulmonary disease (COPD) by reducing airway smooth muscle cell (ASMC) function. It also modifies mTOR activity, microRNA (miR)-101-3p expression, and mitochondria function. Here, the link between miR-101-3p and mTOR-regulated mitochondria integrity and ASMC deterioration was assessed.

**Methods:**

Disease-specific miR-101-3p expression was determined by RT-PCR in primary ASMC (non-COPD smokers: *n* = 6; COPD: *n* = 8; healthy: *n* = 6). The regulatory effect of miR-101-3p modification on mTOR expression, mitochondrial fragmentation, and remodeling properties (*α*-SMA, fibronectin, MTCO2, and p70S6 kinase) was assessed in ASMC (healthy nonsmokers: *n* = 3; COPD: *n* = 3) by Western blotting and immunofluorescence microscopy. MiR-101-3p was modified by specific mimics or inhibitors, in ASMC stimulated with TNF-*α* (10 ng/ml) or cigarette smoke extract (CSE).

**Results:**

MiR-101-3p expression was significantly higher in ASMC of COPD patients, compared to ASMC of healthy or active smokers. MiR-101-3p expression was increased by TNF-*α* or CSE. TNF-*α* or miR-101-3p deteriorated ASMC and mitochondria, while decreasing mTOR signaling, *α*-SMA, fibronectin, and MTCO2. MiR-101-3p inhibition reduced ASMC deterioration and mitochondrial fragmentation.

**Conclusion:**

Constitutive high miR-101-3p expression characterizes COPD-ASMC, causing increased mitochondrial fragmentation and ASMC deterioration. Thus, reactivation mTOR or blocking miR-101-3p presents a potential new strategy for COPD therapy.

## 1. Introduction

Chronic obstructive pulmonary disease (COPD) was the major cause of death of all chronic inflammatory lung diseases in 2017 [1]. According to this latest analysis, the major cause of COPD is cigarette smoking and inhalation of fine dusts. COPD is characterized by irreversible, fixed airflow limitation, and declining forced expiratory volume in 1 second (FEV1). Additional factors such as air pollution, genetic or epigenetic predisposition, and chronic airway infections contribute to the severity of COPD [2, 3]. With the progression of the disease, chronic lung inflammation drives irreversible lung tissue deterioration and tissue remodeling in the small airways, leading to emphysema and chronic bronchiolitis [4].

Tumor necrosis factor alpha (TNF-*α*) plays a crucial role in the pathogenesis of COPD by modifying the function of lung resident tissue forming cell types, including epithelial cells, fibroblasts, and airway smooth muscle cells (ASMC), as well as immune cells [5]. Increased circulating TNF-*α* levels were reported in COPD patients [6]. High serum TNF-*α* levels correlated with the exacerbation frequency, GOLD staging (Global Initiative for Chronic Obstructive Lung Disease), and prediction of FEV1 decline in COPD [7]. TNF-*α* in the sputum distinguished COPD patients from controls [8]. The causative role of TNF-*α* in lung tissue restructuring was supported by animal models, where exposure to cigarette or environmental smoke caused inflammation and remodeling [9–11].

In COPD, TNF-*α* increased monocyte inflammation through mTOR (mammalian target of rapamycin)-regulated glucocorticoid insensitivity [12]. Cigarette smoke-induced pulmonary emphysema was linked to mTOR suppression causing lung injury [13]. In contrast, activation of mTOR-suppressed cigarette smoke induced epithelial cell death and inflammation [14]. In other conditions, TNF-*α* stimulated the expression of microRNA (miR)-101-3p and reduced the expression of mitogen-activated protein kinases (MAPK) [15, 16].

In the lung tissue of COPD patients, miR-101-3p was elevated compared to control tissues specifically in bronchial epithelial cells [17]. Comparing the expression of six different miRs in patients with tuberculosis versus other lung diseases, the expression of miR-101-3p was highest in COPD and lung cancer patients [18]. In non-small-cell lung cancer, the expression of miR-101-3p negatively correlated with drug resistance and epithelial to mesenchymal transition [19]. The expression of miR-101-3p inhibited growth and metastasis of non-small-cell lung cancer cells by interfering with the Akt (serine/threonine kinase or protein kinase B)—mTOR signaling pathway [19]. These data suggest that TNF-*α* might modify the airway and lung tissue structure through miR-101-3p in COPD.

In this study, the effect of TNF-*α* on the expression of miR-101-3p and its contribution to mitochondrial fragmentation was assessed in ASMC obtained from patients with COPD, active smokers without COPD, and nondiseased controls.

## 2. Materials and Methods

### 2.1. Primary Airway Smooth Muscle Cells

Six primary human ASMC lines were purchased from Ruwag (Bettlach, Switzerland). Three cell lines were originated from nonsmoking healthy controls and three from COPD patients. These cells were used for the analysis of cell intracellular signaling; the available patient information is presented in Table 1.

Additional primary ASMC lines were obtained from a local cell bank (Pneumology, University Hospital Basel) collected since 2002. Patients provided informed consent, and the study was approved by the local Ethics Board (Ethic commission of both Basels, Switzerland, EK: 05/06).

Cells were grown in DMEM (Dulbecco's modified Eagle's medium) supplemented with smooth muscle growth supplement (SMGS, #S00725, Thermofisher Scientific, Basel, Switzerland). ASMCs were characterized by the expression of fibrillar *α*-smooth muscle actin (ab5694, Abcam, Cambridge, UK) and negative staining for E-cadherin (#610181, BD Biosciences, Eysins, Switzerland.) as described earlier [20, 21]. All experiments were performed between passages 3 and 8.

### 2.2. Cigarette Smoke Extract (CSE) Preparation

Special standardized research cigarettes (1R6F, University of Kentucky, USA) were used to prepare CSE as previously described [22, 23]. In brief, the cigarettes were smoked via a manual negative pressure syringe. A total of 300 ml of cigarette smoke was bubbled through 10 ml DMEM medium in a glass bottle and mixed by shaking and the pH was adjusted to 7.4. The solution was passed through a 0.22 m filter and defined as 100% CSE. Working concentrations were made by diluting the 100% CSE with culture medium.

### 2.3. Cell Treatment and miR Mimic and Inhibitor Transfection

ASMCs were seeded into 6-well plates (10,000 cells/well) and allowed to adhere for 24 hours before being deprived of serum overnight (DMEM + 0.1% serum, 12 hours). Cells were then exposed to CSE (1% and 5%) for 24 hours or to human recombinant TNF-*α* (10 ng/mL, # 210-TA-005, R&D Systems, Abingdon, UK) for 0, 3, 6, and 24 hours.

MiR-101-3p function was assessed in subconfluent ASMC (70% density). Cells were transiently transfected with either miR-101-3p mimics or inhibitors at concentrations between 5 and 50 nM (#MSY0000099, #MIN0000099, Qiagen, Hombrechtikon, Switzerland), using HiPerfect (#301705, Qiagen) for 48 hours, before being stimulated with TNF-*α*.

### 2.4. Real-Time Quantitative PCR for miR-101-3p Expression

Total RNA was purified by Quick-RNA Mini Prep (#R1055, Zymo Research, Freiburg i. Brsg., Germany). Synthesis of cDNA was performed in 0.5 *μ*g RNA/sample with miR-specific stem loop reverse transcription (RT) using Mir-XTM miRNA First-Strand Synthesis kit (#638315, Clontech, Takara, Göteborg, Sweden). Real-time quantitative PCR (RT-qPCR) was performed by Applied Biosystems 7500 system, FastStart Universal SYBR Green (#04913850001, Roche Diagnostic, Zug, Switzerland), and miR-101-3p expression was normalized to U6 snRNA using MW (2^−ΔΔCT^) algorithms. Primer sequence for miR-101-3p was 5′-TAC AGT ACT GTG ATA ACT GAA-3′ (Microsynth, Basel, Switzerland).

### 2.5. Western Blotting

Proteins were lysed in radio immuno-precipitation assay (RIPA) buffer (#R0278, Sigma-Aldrich, Buchs, Switzerland) containing protease inhibitors (#78447, Thermofisher) and quantified by BCA (#23227, Thermofisher). Equal amounts of denatured protein (20 *μ*g) were size-fractionated in 4–12% SDS-PAGE (#M41212, GeneScript, New Jersey, USA) and transferred onto nitrocellulose membranes (#88018, Thermofisher). Proteins were detected by antibodies from Cell Signaling Technology for total (t)-p70S6 kinase (#2708), phosphorylated (p)-p70S6 kinase (#9205), *α*-smooth muscle actin (*α*-SMA, #19245), and GAPDH (#2118); for fibronectin (#MA5-11981, Thermofisher); and mitochondria cytochrome c oxidase subunit II (MTCO2, #ab79393, Abcam). Protein bands were visualized by HRP on an Azure C300 digital imaging system (Axonlab, Baden, Switzerland).

### 2.6. Confocal Microscopy

ASMCs were seeded onto coverslips and incubated under various conditions for up to 48 hours. Afterwards, ASMCs were fixed (4% formaldehyde, 15 minutes), permeabilized (0.15% Triton-X100, 15 minutes), and blocked (5% bovine serum albumin, BSA, 1 hour). The anticytochrome c antibody (#556432, BD Bioscience) was incubated overnight (4°C), washed, and visualized after incubation (room temperature, 1 hour) with an Alexa488-labeled antibody (#11001, Thermofisher). F-actin was stained with TRIC-phalloidin and nuclei with DAPI. Images were acquired by Nikon Confocal A1 microscope (40 × 1.3NA FI oil objective) under equal laser power and exposure times in 0.24 *μ*m Z-stack intervals. Images were analyzed as Z-stack projection by imaging software FIJI. The percentage of cells with fragmented mitochondria was determined by a blinded observer for 300 cells per cell line [24].

### 2.7. Statistical Analysis

The null-hypothesis was miR-101-3p, and TNF-*α* has no effect on ASMC.

Data are presented as mean ± SEM and were analyzed by unpaired one-way ANOVA and subsequent paired Student's *t*-test. Statistical data analysis was performed by software GraphPad-Prism7 and *P* values < 0.05 were considered as statistically significant.

## 3. Results

### 3.1. Constitutive High miR-101-3p Expression in ASMC from COPD Patients and Its Induction by CSE

**Figure 1:**
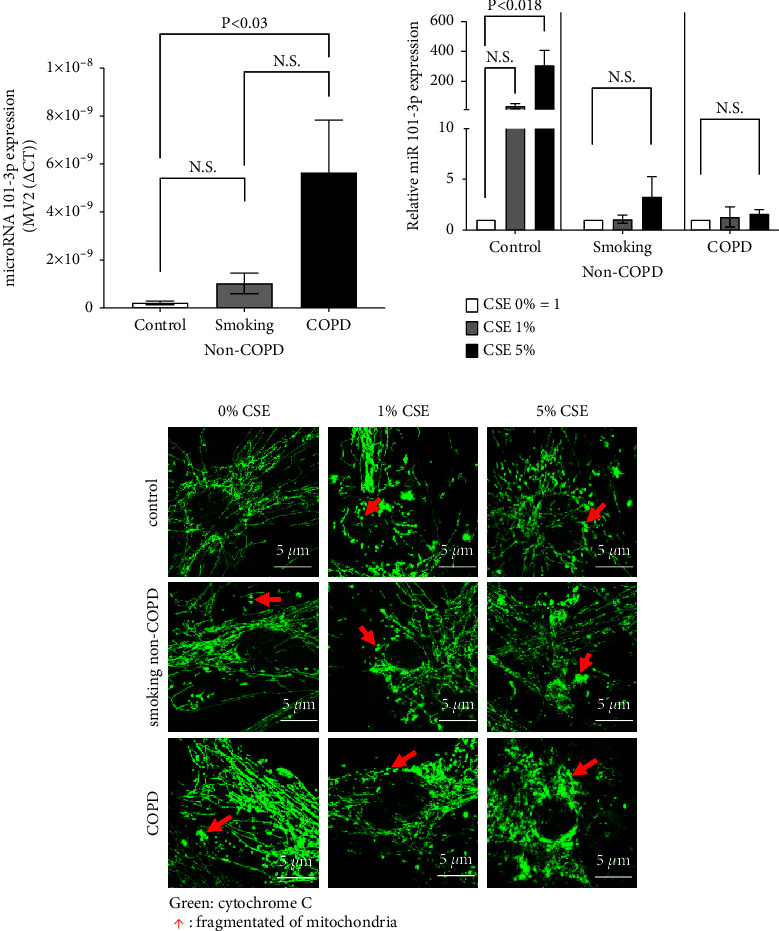
Disease-specific miR-101-3p expression and the effect of cigarette smoke extract (CSE) on ASMC. (a) Disease-specific miR-101-3p gene expressions in unstimulated ASMC from healthy nonsmokers (*n* = 6), active non-COPD smokers (*n* = 6), and COPD patients (*n* = 8). Bars represent the mean ± S.E.M. of the corresponding patient group. (b) Gene expression of miR-101-3p in ASMC exposed for 24 hours to dilutions of CSE. Bars represent the mean ± S.E.M. of independent experiments in three cell lines of each tissue donor group. miR-101-3p expression was compared to untreated (control) ASMC in each group. (c) Z-stack projection photos acquired by confocal microscopy (Nikon, 60x magnification) of fragmented mitochondria morphology after 24 hours of exposure to CSE. Mitochondria are indicated by cytochrome c (green) staining. Similar results were obtained in three additional experiments.

RT-qPCR revealed a significantly higher expression of miR-101-3p in ASMC of COPD patients (*n* = 9, 2^−ΔΔCT^ value: 5.647^−9^ ± 2.188^−9^), when compared to ASMC of nonsmokers (*n* = 6, 2^−ΔΔCT^ value: 2.148^−10^ ± 7.433^−11^), with *P* < 0.05. ASMC of active smokers showed a nonsignificant increased miR-101-3p expression when compared to nonsmokers (*n* = 6, 2^−ΔΔCT^ value: 1.024^−9^ ± 4.287^−10^, *P*=0.920) (Figure 1(a)). In ASMC of nonsmokers, the expression of miR-101-3p was significantly upregulated by CSE at 24 hours, when compared to the basal level of untreated cells (Figure 1(b)). This effect of CSE on miR-101-3p was not significant in ASMC of smokers or COPD patients; however, it has to be considered that the basal expression of smokers and COPD was higher than that of nonsmokers (Figure 1(b)).

Confocal microscopy showed an increase of mitochondrial fragmentation in ASMC of active smokers and COPD patients, when compared to nonsmokers (Figure 1(c), first row). Mitochondrial fragmentation was increased by CSE in a concentration-dependent manner in ASMC of all three groups (Figure 1(c), rows 2 and 3).

### 3.2. MiR-101-3p Modulates Mitochondrial Function

**Figure 2:**
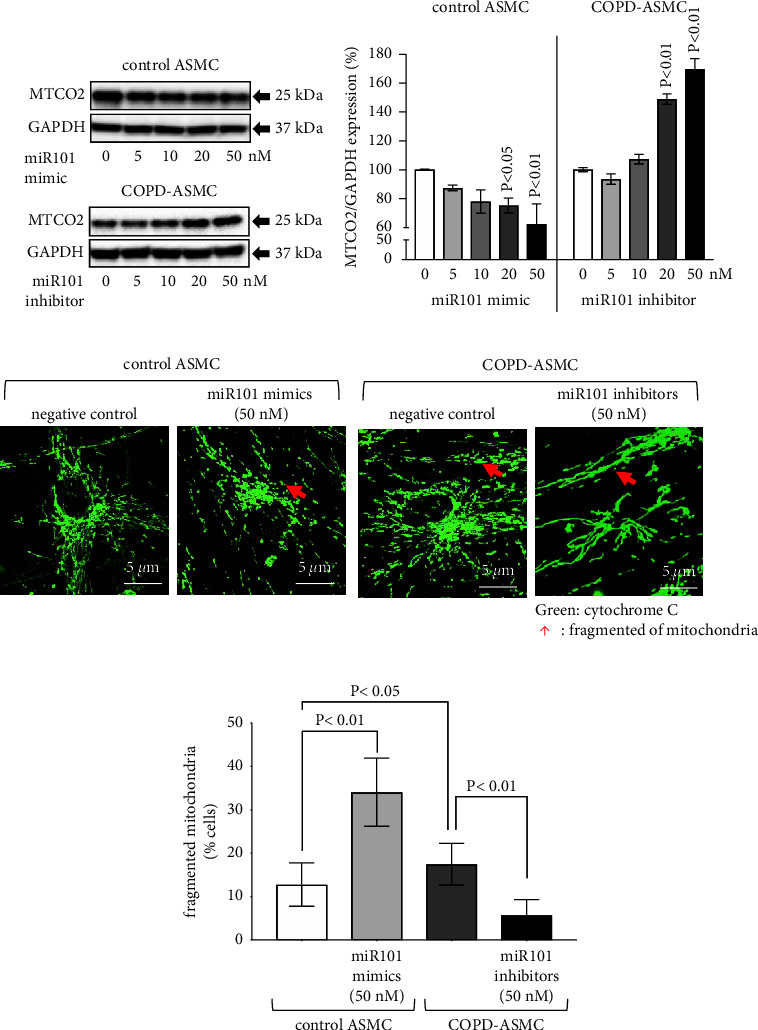
Disease-specific effect of miR-101-3p on ASMC mitochondria. (a) Western blots of mitochondria cytochrome c oxidase subunit II (MTCO2) protein expression in commercial ASMC (healthy nonsmokers (control): *n* = 3; COPD: *n* = 3) treated with miR-101-3p mimics (controls) or inhibitors (COPD). (b) Bars represent the mean ± S.E.M. of MTCO2 expression (image analysis) of three Western blots, as shown in Figure 2(a). *P* values present comparison to untreated cells. (c) Z-stack projection photos acquired by confocal microscopy (Nikon, 60x magnification) of fragmented mitochondria morphology in ASMC of healthy nonsmokers (control, *n* = 3) treated with miR-101-3p mimics, or COPD-ASMC (*n* = 3) treated with miR-101-3p inhibitors, over 24 hours. Mitochondria are visualized by cytochrome c (green) staining. (d) Image analysis of fragmented mitochondria as described in Figure 2(c). Bars represent mean ± S.E.M. of disease-specific effects of miR-101-3p mimics and inhibitors on MTCO2 expression (300 cells/slide).

In ASMC of nonsmokers, transfection with miR-101-3p mimics decreased the expression of the mitochondrial cytochrome c oxidase subunit II (MTCO2) in a concentration-dependent manner as shown by Western blotting and subsequent image analysis (Figures 2(a) and 2(b)). The decreasing effect of miR-101-3p mimics became significant at 20 nM, compared to untreated ASMC (Figure 2(b)). In contrast, treatment of ASMC of COPD patients with miR-101-3p inhibitors upregulated MTCO2 expression in a concentration-dependent manner (Figures 2(a) and 2(b)). The stimulatory effect of miR-101-3p inhibitors in COPD-ASMC became significant at concentration >20 nM (Figure 2(b)).

Transfection with miR-101-3p mimics (50 nM) significantly upregulated mitochondrial fragmentation in ASMC of nonsmokers (Figure 2(c)). In contrast, transfection of COPD-ASMC with miR-101-3p inhibitors (50 nM) significantly reduced mitochondrial fragmentation (Figure 2(c)). The ratio of mitochondrial fragmentation (double blind counting, *n* = 5 per group, 300 cells per experiment) confirmed that miR-101-3p mimics significantly upregulated fragmentation in ASMC of nonsmokers, while miR-101-3p inhibitors significantly downregulated fragmentation in COPD-ASMC (Figure 2(d)).

### 3.3. TNF-*α* Upregulates miR-101-3p Expression and Mitochondrial Fragmentation

**Figure 3:**
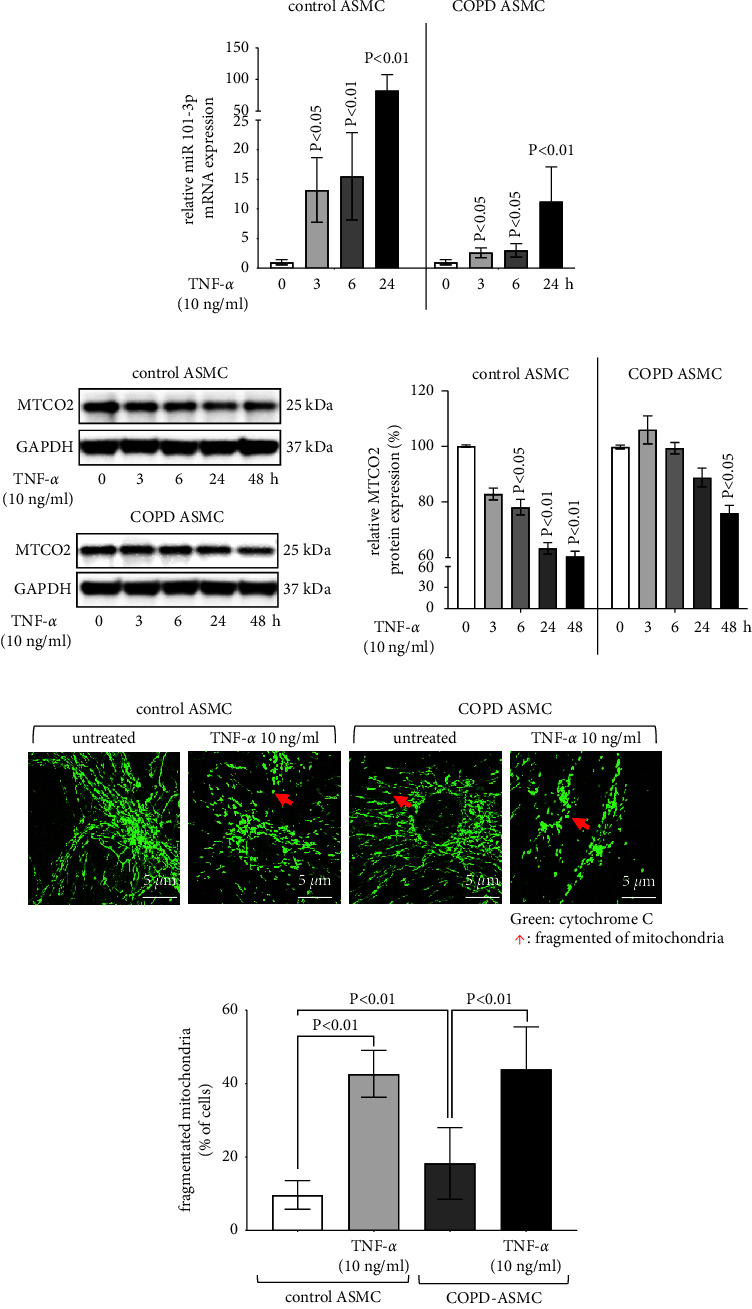
The effect of TNF-*α* on ASMC miR-101-3p expression and mitochondria. (a) Bars show TNF-*α* upregulated miR-101-3p expression in ASMC from healthy nonsmokers (control) and COPD-ASMC (*n* = 3/group). *P* values present comparison to time point 0. (b) Western blots for mitochondrial cytochrome c oxidase subunit II (MTCO2) degradation by TNF-*α* in ASMC of healthy nonsmokers (control, *n* = 3) and COPD-ASMC (*n* = 3). Bars present mean ± S.E.M. of image analysis data. *P* values present comparison to time point 0. (c) Z-stack projection photos (Nikon Confocal, 60x magnification) of mitochondrial fragmentation, induced by TNF-*α* in ASMC of healthy nonsmokers (control) and COPD-ASMC. Mitochondria are indicated by staining of cytochrome c (green). (d) Image analysis of mitochondrial fragmentation is presented in Figure 3(c). Bars represent mean ± S.E.M. of cells with fragmented mitochondria, determined by double blind counting.

TNF-*α* (10 ng/ml) increased miR-101-3p expression and becoming significant at 3 hours in both ASMC of nonsmokers and COPD patients (Figure 3(a)). In ASMC of nonsmokers, TNF-*α* increased miR-101-3p expression by 13.19 ± 2.63 folds at 3 hours, by 15.52 ± 3.26 folds at 6 hours and by 82.52 ± 10.05 folds at 24 hours (Figure 3(a)). In COPD-ASMC, TNF-*α* upregulated miR-101-3p expression by 2.62 ± 0.63 folds at 3 hours, 3.01 ± 0.50 folds at 6 hours and 11.23 ± 3.65 folds at 24 hours (Figure 3(a)). However, it should be noted that the basal miR-101-3p level in COPD-ASMC was significantly higher compared to ASMC of nonsmokers.

In regards to mitochondrial activity, TNF-*α* decreased the basal level of MTCO2 in ASMC of both nonsmokers and COPD patients, as shown by Western blotting and subsequent image analysis (Figure 3(b)). Compared to the basal level, the reducing effect of TNF-*α* was stronger in ASMC of nonsmokers than in COPD-ASMC (Figure 3(b), bar chart). The reducing effect of TNF-*α* on MTCO2 expression was time-dependent and achieved significance after 6 hours in ASMC of nonsmokers, while it only became significant in COPD-ASMC after 48 hours (Figure 3(b)).

Analyzed by confocal microscopy, TNF-*α*-induced mitochondrial fragmentation in ASMC of nonsmokers and COPD patients (Figure 3(c)). Image analysis showed that TNF-*α* significantly increased mitochondrial fragmentation in ASMC of nonsmokers and COPD patients (Figure 3(d)). Furthermore, confocal microscopy confirmed the increased basal level of mitochondrial fragmentation in COPD-ASMC (Figure 3(d)).

### 3.4. TNF-*α*-Induced miR-101-3p Affected ASMC Remodeling

**Figure 4:**
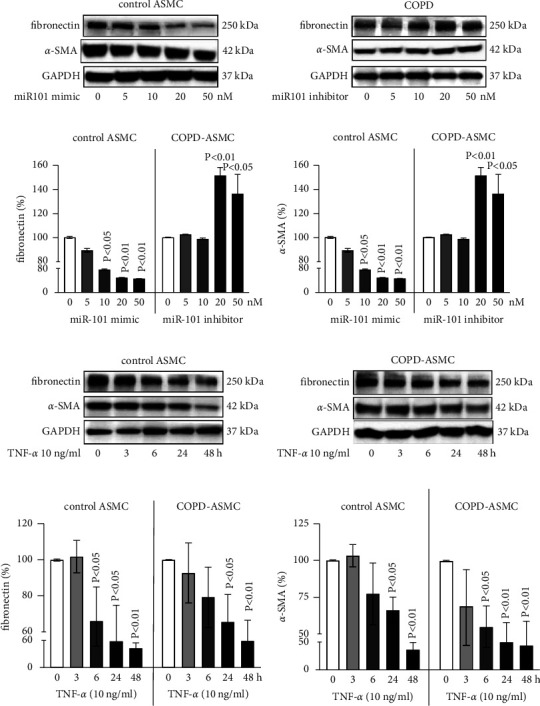
The effect of miR-101-3p and TNF-*α* on ASMC remodeling. (a) Western blots for fibronectin and *α*-SMA protein expression in ASMC of healthy nonsmokers (*n* = 3) treated with miR-101-3p mimics at 48 hours. (b) Western blots for fibronectin and *α*-SMA protein expression at 48 hours in COPD-ASMC (*n* = 3) treated with miR-101-3p mimics. (c) Image analysis of fibronectin expression in ASMC of healthy nonsmokers (control) and COPD, as shown in Figures 4(a) and 4(b). (d) Image analysis of *α*-SMA expression in ASMC of healthy nonsmokers (control) and COPD, as shown in Figures 4(a) and 4(b). (e) Western blots of TNF-*α*-induced fibronectin and *α*-SMA protein expression in ASMC of healthy nonsmokers (control). (f) Western blots of TNF-*α*-induced fibronectin and *α*-SMA protein expression in COPD-ASMC. (g) Image analysis of TNF-*α*-induced fibronectin expression in ASMC of healthy nonsmokers (control) and COPD, as shown in Figures 4(e) and 4(f). (h) Image analysis of TNF-*α*-induced *α*-SMA expression in ASMC of healthy nonsmokers (control) and COPD, as shown in Figures 4(e) and 4(f). Bar charts represent protein expression as mean ± S.E.M. All *P* values represent comparison to untreated cells.

Western blotting showed that ASMC transfection with miR-101-3p mimics decreased fibronectin and *α*-SMA expression in ASMC of nonsmokers concentration dependently (Figure 4(a)). Inhibition of miR-101-3p increased fibronectin and *α*-SMA in a concentration-dependent manner in COPD-ASMC (Figure 4(b)). Image analysis showed that the reducing effect of miR-101-3p mimics on both proteins became significant at concentrations >10 nM in ASMC of nonsmokers (Figures 4(c) and 4(d)). The stimulatory effect of miR-101-3p inhibitors on both proteins became significant at concentrations >20 nM in COPD-ASMC (Figures 4(c) and 4(d)).

Assessed by Western blotting, TNF-*α* time dependently decreased fibronectin and *α*-SMA expression in ASMC of nonsmokers and COPD-ASMC (Figures 4(e) and 4(f)). Image analysis revealed that the effect of TNF-*α* on fibronectin became significant at 6 hours, and further decreased to 30% of the baseline level at 48 hours in ASMC of nonsmokers (Figures 4(g) and 4(h)). In COPD-ASMC, the reducing effect of TNF-*α* on fibronectin expression became only significant at 24 hours (Figure 4(g)), while the expression of *α*-SMA was significantly reduced after 6 hours (Figure 4(h)).

### 3.5. The Effect of miR-101-3p and TNF-*α* Is Regulated via mTOR-p70S6K Signaling

**Figure 5:**
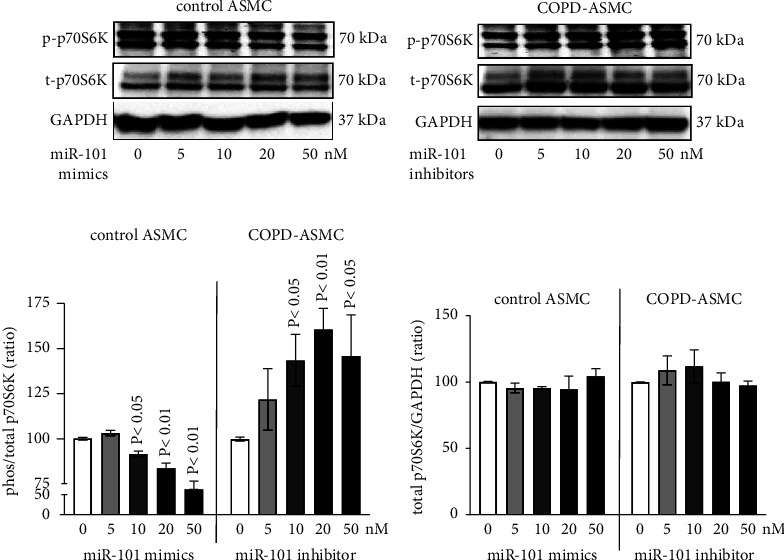
The effect of miR-101-3p on mTOR signaling pathway. (a) Concentration-dependent modification of phosphorylation (p-)p70S6K by miR-101-3p mimics in ASMC of healthy nonsmokers (control, *n* = 3). (b) Concentration-dependent modification of phosphorylation (p-)p70S6K by miR-101-3p inhibitors in COPD-ASMC (*n* = 3). (c) Comparison of the p-/t- p70S6K ratio in five Western blots of ASMC from healthy nonsmokers (control) to COPD by image analysis (Figures 5(a) and 5(b)). Bars represent mean ± S.E.M. *P* values were calculated by comparison to untreated cells. (d) Comparison of the t-p70S6K/GAPDH ratio in five Western blots in ASMC from healthy nonsmokers (control) to COPD by image (Figures 5(a) and 5(b)). Bars represent mean ± S.E.M.

Transfection with miR-101-3p mimics decreased the phosphorylation of p70S6K (p-p70S6K) in all ASMC, shown by Western blotting (Figure 5(a)). In contrast, transfection with miR-101-3p inhibitors increased the p70S6K phosphorylation at 48 hours (Figure 5(b)). Based on image analysis of all Western blots, these effects achieved significance at concentrations >20 nM for miR-101-3p mimics, and >10 nM for miR-101-3p inhibitors (Figure 5(c)). The expression of total (t-)p70S6K was not affected by any miR-101-3p modifiers (Figure 5(d)).

**Figure 6:**
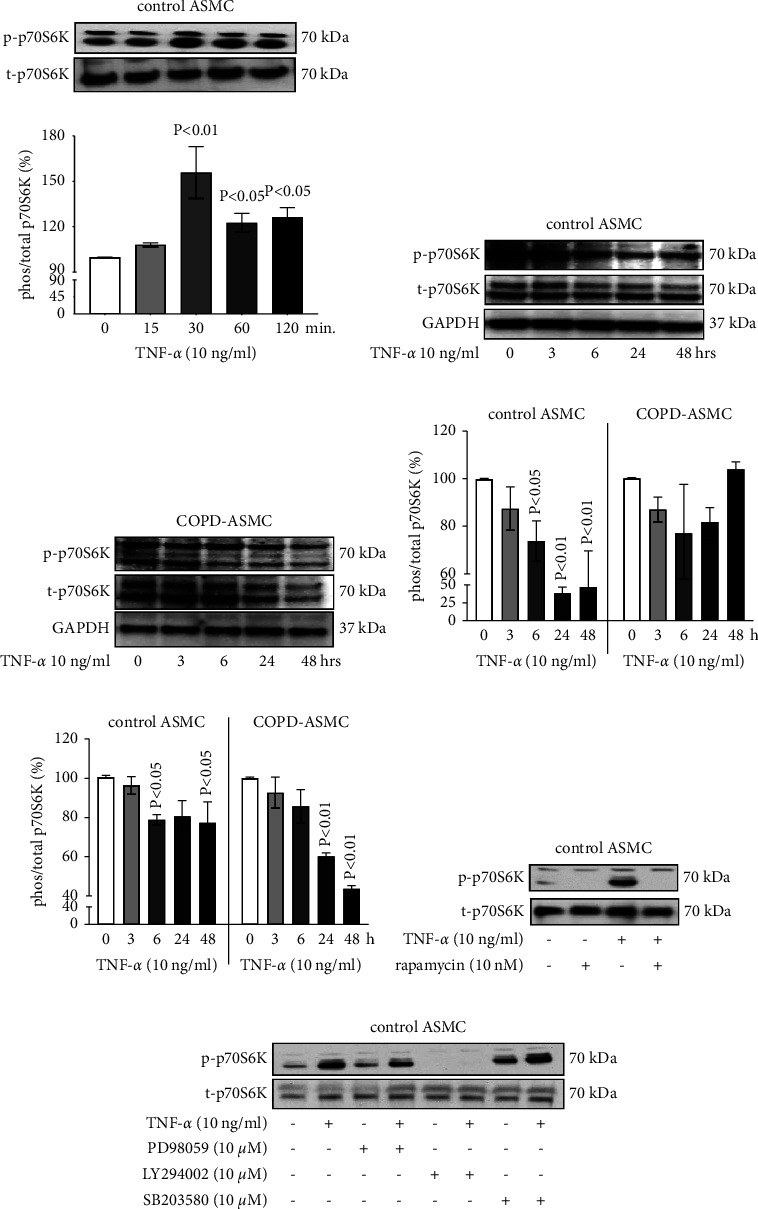
The effect of TNF-*α* on mTOR signaling pathway. (a) Western blots of TNF-*α*-induced short-term p70S6K phosphorylation (120 min.) in healthy nonsmokers (*n* = 3) and subsequent image analysis. Bars show the ratio of phosphorylated (p-) to total (t)-p70S6K as percentage of time point 0. (b) Western blots of TNF-*α*-stimulated long-term (48 hours) phosphorylation of (p)-p70S6K in ASMC of healthy nonsmokers (control, *n* = 3). (c) Western blots of TNF-*α*-induced long-term p70S6K phosphorylation in COPD-ASMC (*n* = 3) over 48 hours. (d) Image analysis of Western blots is presented in Figures 6(b) and 6(c). Bars compare the ratio of phosphorylation (p)-p70S6K to total (t-) p70S6K. (e) Image analysis of Western blots is presented in Figures 6(b) and 6(c). Bars show the ratio of t70S6K to GAPDH as percentage of time point 0. (f and g) Representative Western blots of TNF-*α*-induced p70S6K phosphorylation in ASMC of healthy nonsmokers (control) in the presence of different signaling pathway inhibitors (Rapamycin, PD98059, LY294002, and SB203580) at 24 hours. Similar results were obtained in three additional experiments. All *P* values were calculated by comparison to untreated cells.

The TNF-*α*-induced phosphorylation of p70S6K returned to the baseline level after 120 minutes (Figure 6(a)). However, when treated with TNF-*α* over 48 hours, a disease-specific effect was observed. In ASMC of nonsmokers, but not COPD-ASMC, TNF-*α* significantly decreased the phosphorylation of p70S6K over time (Figures 6(b)–6(d)). TNF-*α* had a weaker reducing effect on total p70S6K expression in ASMC of nonsmokers, but time dependently and significantly reduced total p70S6K levels in COPD-ASMC (Figure 6(e)). Chemical inhibitors of the mTOR signaling pathway, rapamycin, were used to confirm the involvement of TNF-*α*-induced mTOR signaling on p70S6K phosphorylation (Figures 6(f) and 6(g)).

### 3.6. The Additive Effect of TNF-*α* and miR-101-3p on ASMC Deterioration

**Figure 7:**
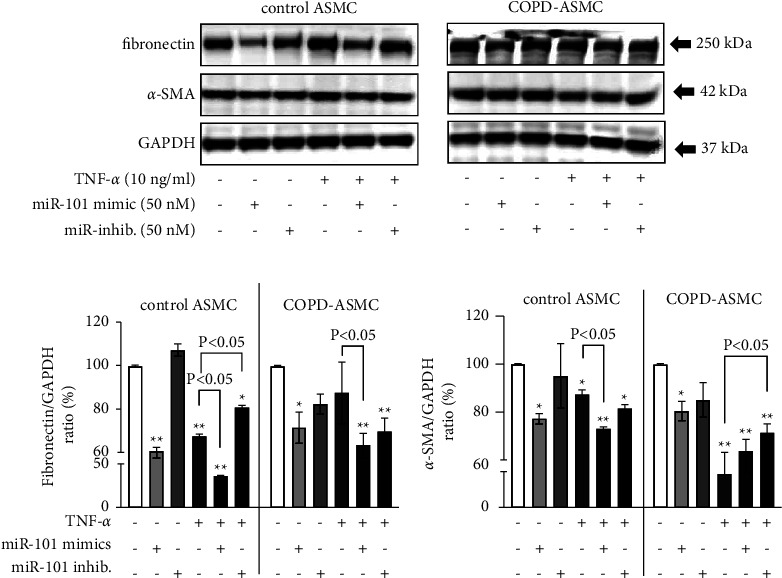
The additive effect of miR-101-3p and TNF-*α* on ASMC deterioration. (a) Western blots of disease-specific 24 hours effect for miR-101-3p mimics and inhibitors on the baseline expression of fibronectin and *α*-SMA expression in AMSC of healthy nonsmokers (*n* = 3) and COPD (*n* = 3). (b) Image analysis of fibronectin Western blots in ASMC of healthy nonsmokers and COPD, as shown in Figure 7(a). (c) Image analysis of *α*-SMA Western blots in ASMC of healthy nonsmokers and COPD, as shown in Figure 7(a). Bars represent mean ± S.E.M. “^*∗*^” = *P* < 0.05 and “^*∗∗*^” = *P* < 0.01 are compared to untreated ASMC.

In ASMC of nonsmokers, miR-101-3p mimics modified the expression of fibronectin and *α*-SMA in the presence and absence of TNF-*α* (Figure 7(a)). TNF-*α* reduced the baseline expression of fibronectin significantly in ASMC of nonsmokers, but not in cells of COPD patients as shown by Western blotting and subsequent image analysis (Figures 7(a) and 7(b)). MiR-101-3p mimics alone reduced the base line expression of fibronectin significantly in both ASMC of nonsmokers and COPD patients (Figures 7(a) and 7(b)). When combining TNF-*α* with miR-101-3p mimics, the inhibition of fibronectin was additive in ASMC of nonsmokers, but not in COPD-ASMC (Figures 7(a) and 7(b)).

TNF-*α* significantly reduced the base line expression of *α*-SMA in ASMC of nonsmokers and COPD patients (Figures 7(a) and 7(c)). MiR-101-3p mimics alone reduced the baseline expression of *α*-SMA in ASMC of both nonsmokers and COPD patients (Figure 7(c)). Pretreatment with miR-101-3p mimics further reduced *α*-SMA expression in TNF-*α*-treated cells of nonsmokers, but not in COPD-ASMC (Figures 7(a) and 7(c)). Preincubation with miR-101-30 inhibitors had no significant effect on base line *α*-SMA, but significantly compensated the suppression by TNF-*α* in COPD-ASMC (Figure 7(c)).

## 4. Discussion

This study provided evidence that miR-101-3p is constitutively upregulated by over100 folds in ASMC of COPD patients when compared to cells of smokers and healthy controls. This suggests an intrinsic lasting effect of cigarette smoking on miR-101-3p regulation. Increasing miR-101-3p levels reduced mTOR signaling, which impaired mitochondrial function. Consequently, the expression of *α*-SMA and fibronectin was reduced.

COPD is one of the most prevalent chronic inflammatory lung diseases worldwide and is responsible for significant morbidity and premature mortality (GOLD report 2019, https://goldcopd.org/gold-reports/). In COPD, the remodeling of small airways is a main pathology, and results in the irreversible decline of lung function [3, 4]. The destruction of the COPD lung tissue manifests as the breakdown of the small airways and alveolar wall attachment without fibrosis. These events lead to airspace enlargement, hyperinflation, and loss of pulmonary plasticity [25]. During the progression of COPD, small conducting airways disappear, before the emphysematous destruction of the parenchymal lung tissue [26]. In advanced emphysema, dysfunctional ASMC further contribute to ventilation-perfusion (V/Q) inequality, and increased exacerbations [27, 28].

TNF-*α* is highly expressed in lung tissues and the body fluids of COPD patients [6–8]. Inflammatory cells and structural cells of the small airways of COPD patients responded to proinflammatory proteins including interleukin (IL)-1*β*, IL-6, TNF-*α*, and TGF-*β* [29]. ASMCs are a key cell type of the airway wall and contribute to the pathogenesis of COPD by acting as target cells and as initiators of chronic inflammation and remodeling [30, 31]. In an animal model, cigarette smoke upregulated miR-101-3p expression in the COPD lung tissue and in bronchial epithelial cells [17]. In this study, the increased expression of miR-101-3p was maintained in isolated ASMC of COPD patients, but not in ASMC of controls or active smokers. The data presented above suggest that (i) the constitutive expression of miR-101-3p in COPD becomes independent from the exposure to cigarette smoke and is maintained in isolated ASMC; (ii) cigarette smoking alone is insufficient to imprint the high expression of miR-101-3p. Thus, future studies are needed to identify the cause of such event. However, the presented data suggest that TNF-*α* played the role in the upregulation of miR-101-3p, as it has been suggested in other conditions [32].

TNF-*α* was described as the main initiator or mediator of cigarette smoke-induced emphysema and therefore contributed to the degradation of the lung tissues in COPD [33]. In regards to ASMC remodeling, TNF-*α* and the mTOR signaling pathway played a central role of the *de novo* synthesis and the degradation of extracellular matrix components such as collagens and fibronectin [34]. Here, we showed that in ASMC from controls, the expression of miR-101-3p was induced by TNF-*α*, while in COPD-ASMC, such a stimulatory effect was not observed; most likely due to the fact that the miR-101-3p level was over 100 times higher compared to healthy controls and non-COPD smokers. The literature indicates that miR-101-3p is a negative regulator of mTOR signaling, and thereby prevented autophagy [35]. This observation is in line with the downregulation of mTOR signaling by TNF-*α* in ASMC shown in this study. In COPD cells, TNF-*α* had no significant effect on the phosphorylation of p70S6K, while it reduced the phosphorylation of nonsmokers. This observation suggests a deregulation of TNF-*α* signaling in COPD. However, others reported that activation of mTOR corrected mitochondrial fission and mitophagy in other cell types [36]. These opposing functions of mTOR on mitochondrial regulation might be disease specific and needs to be further investigated.

Mitochondrial dysfunction is a central pathology in various chronic inflammatory lung diseases [37, 38]. Infection, cigarette smoke, and environmental insults are known to affect mitochondria mass and mitochondrial activity. Mitochondria contributed to airway diseases by aberrant energy metabolism, excessive reactive oxidative species production, intracellular calcium overload, and decreased compensatory capacities to stress [37, 38]. The function and structure of mitochondria was regulated by mTOR signaling [39, 40]. In an earlier study on asthma, mTOR signaling increased mitochondrial function in ASMC and thereby controlled remodeling [41]. In this study, the inhibition of mTOR signaling by TNF-*α* led to mitochondrial fragmentation and cell deterioration in COPD-ASMC. The inhibition of the mTOR signaling by TNF-*α* also reflected in a significant decrease of MTCO2, leading to mitochondrial fragmentation in isolated COPD-ASMC. The same effects on mitochondria were induced by upregulation of miR-101-3p. Furthermore, the suppression of p70S6K, a key mTOR protein, resulted in mitochondrial fragmentation and ASMC deterioration. In contrast, miR-101-3p inhibition had the opposing effects.

This investigation has some limitations: the subject numbers were relatively small due to limited access to donor samples, the existence of a possible cell type difference for the above described effects of TNF-*α* and miR-101-3p was not investigated, and the increased expression of miR-101-3p was not confirmed in COPD tissue sections, due the lack of specially prepared tissue samples. Furthermore, the study did not compare if the upregulation of miR-101-3p correlated with the GOLD staging of COPD or if it occurred in nondiseased smokers. These topics need future investigations.

The presented data indicate a COPD-specific constitutively increased expression of miR-101-3p in ASMC isolated from COPD patients impairs mTOR signaling and leads to mitochondrial fragmentation-dependent ASMC deterioration. Reactivating mTOR or blocking miR-101-3p presents a potential new strategy for COPD therapy.

## Figures and Tables

**Table 1:** Clinical parameters of primary cell donors.

Diagnosis	Age	Gender	Smoking status	FEV1%
Control	66	Male	Nonsmoker	89
Control	65	Female	Nonsmoker	89
Control	78	Male	Nonsmoker	102
Control	78	Female	Nonsmoker	108
Control	75	Male	Nonsmoker	97
Control	75	Male	Nonsmoker	98

Smoker	56	Male	Current smoker	NA
Smoker	74	Male	Current smoker	NA
Smoker	42	Male	Current smoker	NA
Smoker	82	Male	Current smoker	NA
Smoker	44	Male	Current smoker	NA
Smoker	56	Male	Current smoker	94

COPD	74	Female	20 PY	76
COPD	68	Female	NA	41
COPD	64	Male	40 PY	72
COPD	60	Male	Active	55
COPD	71	Male	25 PY	35
COPD	76	Male	NA	22
COPD	53	Male	39 PY	64
COPD	70	Male	70 PY	34

FEV1: forced expiratory volume over 1 second. PY: pack years.

## Data Availability

The original data are available on request from the first author.
